# Boosting Photocatalytic Water Splitting of Polymeric C_60_ by Reduced Dimensionality from Two-Dimensional Monolayer to One-Dimensional Chain

**DOI:** 10.1021/acs.jpclett.3c02578

**Published:** 2023-12-21

**Authors:** Cory Jones, Bo Peng

**Affiliations:** † Selwyn College, University of Cambridge, Grange Road, Cambridge CB3 9DQ, United Kingdom; ‡ Theory of Condensed Matter Group, Cavendish Laboratory, University of Cambridge, J. J. Thomson Avenue, Cambridge CB3 0HE, United Kingdom

## Abstract

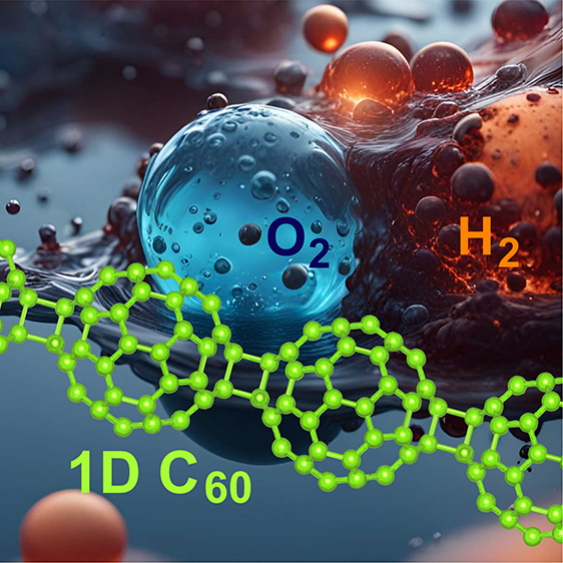

The recent synthesis of monolayer fullerene networks (HouL., et al. Nature2022, 606, 50735705817 10.1038/s41586-022-04771-5) provides new opportunities for photovoltaics and photocatalysis because of their versatile crystal structures for further tailoring of electronic, optical, and chemical function. To shed light on the structural aspects of the photocatalytic water splitting performance of fullerene nanomaterials, we compare the photocatalytic properties of individual polymeric fullerene chains and monolayer fullerene networks from first-principles calculations. We find that the photocatalytic efficiency can be further optimized by reducing the dimensionality from two-dimensional (2D) to one-dimensional (1D). The conduction band edge of the polymeric C_60_ chain provides an external potential for the hydrogen reduction reaction much higher than that of its monolayer counterparts over a wider range of pH values, and there are 2 times more surface active sites in the 1D chain than in the 2D networks from a thermodynamic perspective. These observations identify the 1D fullerene polymer as a more promising candidate as a photocatalyst for the hydrogen evolution reaction in comparison to monolayer fullerene networks.

In photocatalytic water splitting, water is decomposed into hydrogen and oxygen using light, which can produce hydrogen as a green energy alternative to fossil fuels.^1−13^ Recently synthesized two-dimensional (2D) fullerene networks^14,15^ hold great promise for such applications owing to the benefits of (1) a suitable band gap to generate a large amount of electron–hole pairs, (2) a high carrier mobility for separating electrons and holes, and (3) appropriate band edges for thermodynamically driving the hydrogen evolution reaction with the help of photoexcited electrons, and the computational predictions based on such microscopic mechanisms^16^ have been recently confirmed experimentally.^17^ Among the different structural phases of polymeric C_60_ monolayers, the quasi-one-dimensional quasitetragonal phase (qTP1) and the tightly bound quasitetragonal phase (qTP2) have enhanced photocatalytic water splitting performance compared to that of the quasihexagonal phase (qHP). However, monolayer qTP2 C_60_ is thermodynamically less stable than monolayer qTP1 C_60_ at room temperature, while monolayer qTP1 C_60_ tends to split into individual one-dimensional (1D) chains because of its low dynamic and mechanical stability.^18^ Therefore, it is worth investigating the electronic, optical, transport, and thermodynamic properties of the 1D fullerene polymer in the context of the photocatalysis of the hydrogen evolution reaction.

The theoretical prediction and experimental synthesis of the 1D fullerene polymer date to the 1990s.^19−22^ In a 1D fullerene chain, each C_60_ cage connects neighboring cages through covalent [2+2] cycloaddition bonds, which can be formed between isolated C_60_ molecules as a result of photo- or pressure-induced polymerization.^23−25^ The 1D polymeric C_60_ chain can be viewed as the building block for a variety of ordered or disordered 1D, 2D, and three-dimensional (3D) fullerene/fullerite polymers^26,27^ with distinct electronic, optical, and vibrational properties.^28−32^ Additionally, polarons and self-trapped excitons can be formed in the 1D C_60_ crystals, resulting in Jahn–Teller distortion that can be described by the Su–Shriffer–Heeger model.^33,34^ The physical and chemical properties of 1D polymeric C_60_ chains can be further tuned by doping, leading to various applications such as superconductivity.^35−38^ However, it is unclear whether 1D fullerene polymers can be assessed as a possible photocatalyst for the hydrogen evolution reaction. In particular, the physical and chemical implications of reducing the dimensionality from 2D to 1D C_60_ on photocatalysis have not yet been investigated.

In this work, we investigate the band structures of the 1D C_60_ chain and 2D qTP2 C_60_ networks using the unscreened hybrid functional, which accurately describes the effects of reduced dimensionality. Due to the similarities in their structures, a direct comparison between 1D C_60_ and 2D qTP2 C_60_ polymers is possible. The excitonic and transport properties of polymeric fullerene chains are also investigated to understand whether electrons and holes can be separated effectively upon photoexcitation. Finally, the free energy barrier for the intermediates in the hydrogen evolution reaction is calculated for all possible adsorption sites, which further confirms that 1D C_60_ has a much higher photocatalytic efficiency owing to twice the number of energetically favored adsorption sites and larger surface area compared to those of their monolayer counterpart.

Figure 1 shows the crystal structures of polymeric fullerene chains and monolayer qTP2 fullerene networks. Both structures have inversion symmetry and are mirror symmetric with respect to the (100), (010), and (001) planes, with *C*_2_ rotational symmetry along the [100], [010], and [001] axes. For 1D C_60_, each C_60_ cage is connected by the in-plane [2+2] cycloaddition bonds along the *a* direction; in 2D qTP2 C_60_, the C_60_ cages along the *a* and *b* directions are linked by the vertical and in-plane [2+2] cycloaddition bonds, respectively. Structural relaxation leads to the following lattice constants: for 1D C_60_, *a* = 9.062 Å, and for 2D qTP2 C_60_, *a* = 9.097 Å and *b* = 9.001 Å.

**Figure 1:**
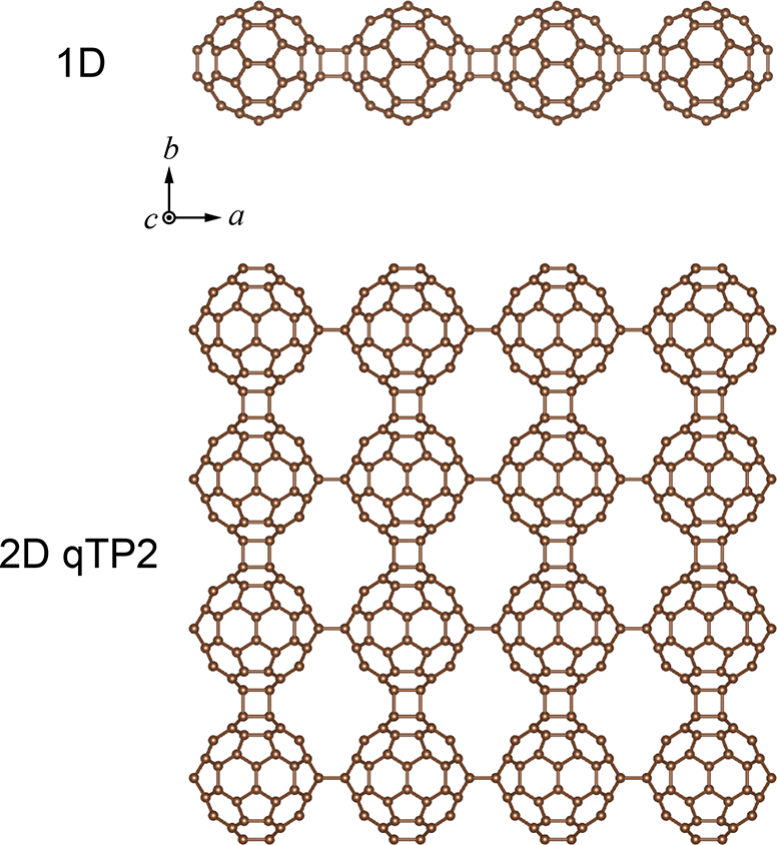
Top views of the crystal structures of 1D and 2D C_60_.

The band structure calculations using the unscreened hybrid functional give band gaps of 2.46 and 2.13 eV for 1D and 2D C_60_, respectively. The larger band gap of the 1D chain can be attributed to the much weaker screening effects, as the dimensionality is reduced from a 2D monolayer to a 1D chain. Compared to the band gap difference of ∼0.6 eV between monolayer and bulk MoS_2_,^39^ the relatively small band gap difference between 1D and 2D C_60_ of 0.33 eV is due to their similar dielectric screening. Static dielectric function ε_∞_ of bulk MoS_2_ is >3.5 times larger than that of its monolayer counterpart, whereas the ε_∞_ of monolayer qTP2 C_60_ is ∼1.5 times the ε_∞_ of 1D C_60_ chains (for details, see the dielectric function in the Supporting Information). As a result, the band gaps of both 1D and 2D C_60_ are close to the band gap of 2 eV required for efficient photocatalysis.^40−42^

Another requirement for photocatalysts is that the band edges must accommodate the redox potentials involved in the water splitting reaction. The calculated band edges for both systems are shown in Figure 2, with the vacuum levels calculated by averaging the electrostatic potential as references. 1D C_60_ has a valence band maximum (VBM) at −6.30 eV and a conduction band minimum (CBM) at −3.84 eV with respect to the vacuum level. Both the CBM and the VBM lie at the X point in the Brillouin zone, resulting in a direct band gap. On the contrary, 2D qTP2 C_60_ has an indirect band gap with the VBM at Γ and the CBM at Y. The VBM of 2D C_60_ at −6.08 eV is 0.22 eV higher than that of 1D C_60_, and its CBM at −3.95 is lower than that of 1D C_60_ by 0.11 eV.

**Figure 2:**
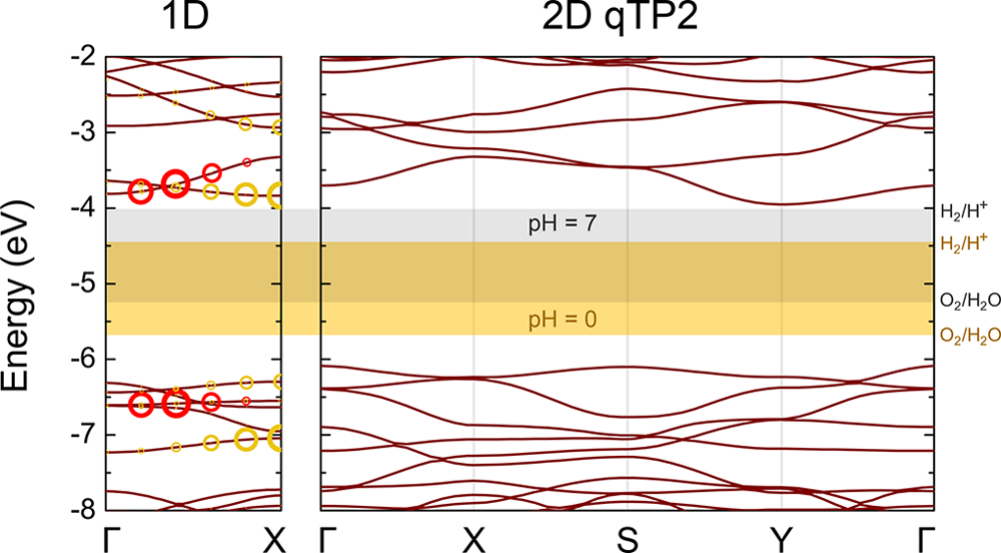
Band structures of the 1D fullerene chain and 2D fullerene networks computed using the unscreened hybrid functional. The red and yellow circles drawn on the 1D band structures indicate the contributions of the corresponding electron–hole pairs to the two brightest excitons near the band edges. The radii of these circles represent their corresponding oscillator strength.

The redox potentials of the relevant half-reactions for pH values of 0 and 7 are also plotted in Figure 2. The CBM of the 1D fullerene polymer is 0.60 eV above the reduction potential of the H_2_/H^+^ half-reaction at a pH of 0, and it reaches the reduction potential at a pH of 10. In monolayer qTP2 C_60_, the reduction potential of the H_2_/H^+^ half-reaction is lower than the CBM at a pH of 7. This renders both 1D and 2D C_60_ polymers suitable for photocatalysis of the hydrogen evolution reaction. However, at a pH of 8, photocatalysis is no longer activated in 2D C_60_, whereas the 1D fullerene polymer can still catalyze the reaction until the pH reaches 10. Therefore, the 1D fullerene polymer can function as an effective catalyst over a greater range of pH conditions than the 2D qTP2 monolayer.

The first step in photocatalysis is the photoexcitation of the catalyst to generate electron–hole pairs. In this process, it is preferable that the optical absorption be strong enough to generate a large number of electron–hole pairs, while the excitonic effects are not too strong so the electrons and holes can be separated effectively. The time-dependent Hartree–Fock calculations of excitonic effects reveal the two brightest excitons (with the largest oscillator strength) near the band edges in 1D C_60_, and the electron–hole pair contributions are illustrated in Figure 2 as circles, the radii of which reflect the contribution of a particular electron–hole pair to the brightest excitons. The two excitons have eigenenergies of 2.57 and 2.77 eV, corresponding to the red and yellow circles, respectively, in Figure 2. The binding energies of the excitons are given by the difference between the independent particle hybrid functional eigenenergy and the exciton eigenenergy. The binding energies of the two brightest excitons are 157 and 44 meV, respectively. Because the holes that contribute the most to the excitons are in much lower valence states than the VBM, carrier thermalization always leads to the dissociation of the excitons. This process gives rise to effective electron–hole separation, and as a result, both the electrons and the holes can proceed to involve themselves in their respective redox reactions.

To investigate whether the electrons and holes can transfer efficiently after the dissociation of the excitons following carrier thermalization, the carrier mobility of 1D and 2D C_60_ is computed at different doping concentrations, as shown in Figure 3. The carrier mobility shows similar trends with increasing concentrations for both systems, as the ionized impurity scattering becomes stronger with more dopants. However, even when the doping concentration is relatively high, i.e., at 10^–3^*e*/formula unit (charge per unit cell), good carrier mobility can still be obtained, which can be attributed to the delocalized π bonds formed near the band edges.^16^

**Figure 3:**
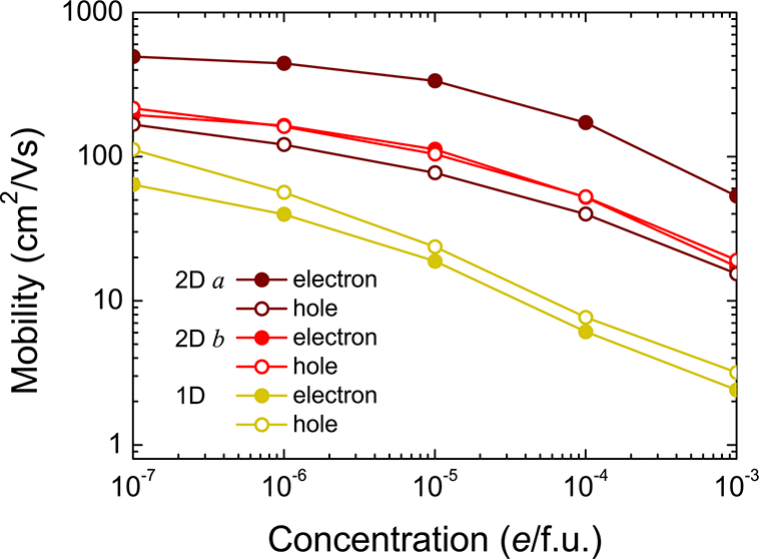
Carrier mobility of 1D and 2D C_60_ at different doping concentrations.

As the electrons and holes are separated, they can participate in their respective half-reactions. The hydrogen evolution half-reaction requires two steps. (1) The photoexcited electron in the conduction band combines with the proton adsorbed on the surface of the catalyst, forming reaction intermediate *H. (2) The hydrogen atoms adsorbed on the surface of the catalyst form diatomic hydrogen. The energy barrier posed by the intermediates is an important factor in the catalysis process.^2,3^ The total change in Gibbs free energy is shown in Figure 4a.

**Figure 4:**
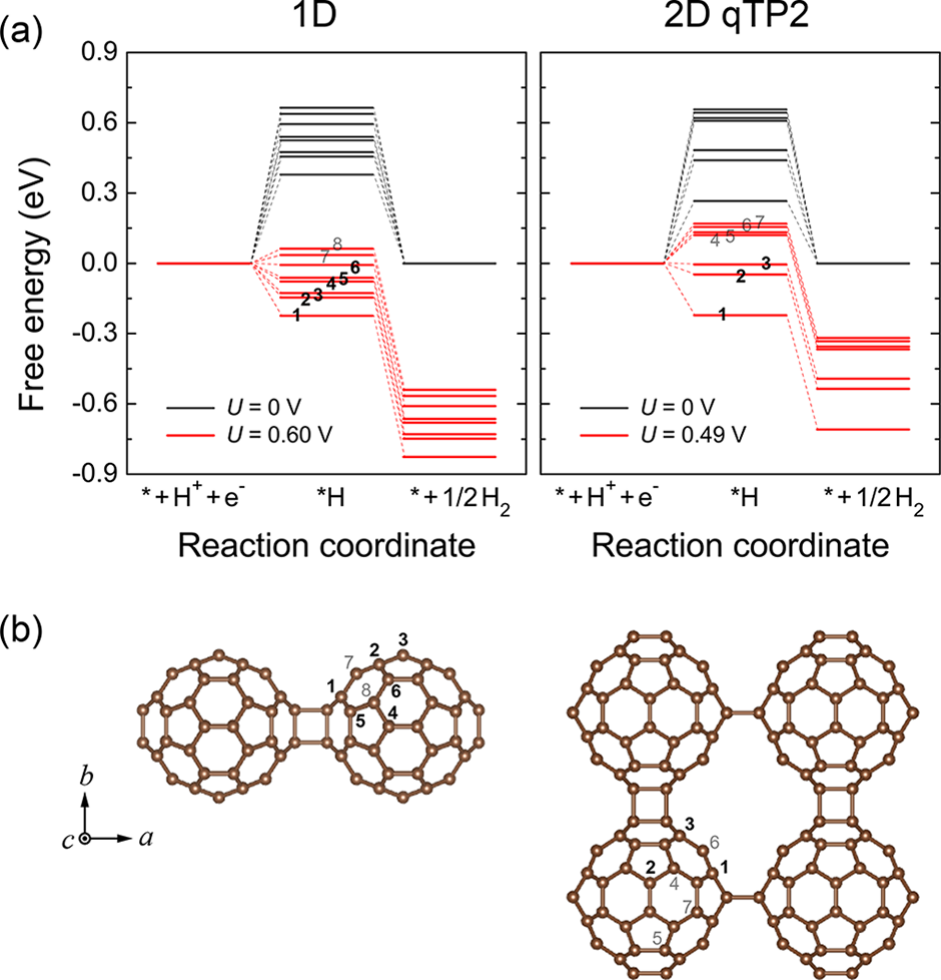
(a) Gibbs free energy changes associated with the hydrogen evolution reaction in the 1D fullerene chain and 2D fullerene networks at a pH of 0 and room temperature, showing the energy barrier posed by the intermediate adsorbate and the effect of photoexcitation in creating a more favorable Gibbs energy in both the intermediates and the products. (b) Adsorption sites for both systems, with lower to higher numbers corresponding to lower to higher free energies of the intermediates, respectively.

There are two paths shown in Figure 4a with zero and finite external potential *U* (black and red, respectively). The *U* = 0 reaction path in black corresponds to catalysis without photoexcitation. From the free energy diagram, one can see that without photoexcitation to provide an external potential, the production of diatomic hydrogen via the hydrogen evolution reaction described above is not spontaneous; i.e., the energy barrier posed by the intermediate is not negative as is suitable for effective catalysis.

The other path of a non-zero potential *U* in red indicates that the photoexcited electron lowers the free energy barrier by *U*. With photoexcitation and the production of electron–hole pairs, electrons in the conduction band can be at a sufficiently high energy for the reaction to become spontaneous. During the reaction, the photoexctied electrons combine with protons to form reaction intermediate *H. In 1D C_60_, an external potential *U* of 0.60 eV is generated by the difference between the CBM and the reduction potential of the H_2_/H^+^ half-reaction, and the free energy change of the intermediate becomes negative for six adsorption sites. In 2D C_60_, *U* = 0.49 eV upon photoexcitation, and the free energy change becomes negative for only three adsorption sites at a pH of 0 and room temperature.

For the 1D polymer, the lowest-energy intermediate is that for which the hydrogen atom is adsorbed on the carbon atom that is nearest to the [2+2] cycloaddition bonds. The energy profiles of the reaction involving each of the symmetry irreducible adsorption sites in the 1D polymer in Figure 4a correspond to the numbers in Figure 4b, with lower numbers indicating lower Gibbs free energies of the intermediates. Only two adsorption sites among eight symmetry irreducible carbon atoms are not thermodynamically favorable, as marked by 7 and 8 in light gray in Figure 4b.

For the 2D polymer, only three adsorption sites of seven are energetically favorable for the hydrogen evolution reaction, with two of them being the nearest neighboring carbon atoms to the [2+2] cycloaddition bonds. Additionally, the four remaining sites, marked by 4–7 in light gray in Figure 4b, have much larger energy barriers of at least 0.12 eV, which are much larger than the thermal fluctuation energy *k*_B_*T* at room temperature (0.026 eV) and are therefore not thermally accessible.

The existence of twice the number of energetically favorable adsorption sites in the 1D chain suggests a greater reaction rate, which renders the 1D polymeric fullerene chain a promising candidate as a photocatalyst for the hydrogen evolution reaction. Additionally, the 1D chain is thermodynamically more stable than monolayer qTP2 fullerene networks,^18^ indicating that the 1D fullerene polymer is a better candidate than the 2D qTP2 monolayer as a photocatalyst.

In conclusion, the electronic structures, excitonic effects, transport properties, and thermodynamics for the hydrogen evolution reaction of 1D fullerene polymers are investigated in the context of its application as a photocatalyst compared with monolayer fullerene networks. We find that reducing the dimensionality from monolayer to chain boosts the photocatalytic performance of polymeric fullerene. The band structures show that the band edges of 1D C_60_ accommodate the reduction potentials of the hydrogen evolution reaction over a wider range of pH conditions than 2D qTP2 C_60_. Time-dependent Hartree–Fock calculations on excitonic effects indicate the effective separation of electron–hole pairs in 1D polymeric fullerene chains upon carrier thermalization, and such separation can further be enhanced by the high carrier mobility. The calculated Gibbs free energy also demonstrates that the 1D fullerene polymer can function as a photocatalyst of the hydrogen evolution reaction on 2 times more adsorption sites than the 2D qTP2 monolayer, as photoexcitation in 1D C_60_ sufficiently reduces the energy barrier posed by the reaction intermediate. Overall, 1D C_60_ can be a much better photocatalyst with higher efficiency and more thermodynamic stability than monolayer C_60_.

## Methods

All calculations are performed using *ab initio* methods as implemented by vasp.^43,44^ The projector-augmented wave (PAW) potential is used^45,46^ with the PBEsol exchange-correlation functional under the generalized gradient approximation (GGA).^47^ A plane wave cutoff of 800 eV is used with **k**-meshes of 5 × 1 × 1 and 5 × 5 × 1 for 1D and 2D C_60_, respectively. Both the lattice constants and the internal coordinates are fully relaxed, until the energy is converged at 10^–6^ eV for the self-consistent loop and the force is converged at 10^–2^ eV/Å for the geometry optimization. A 1D fullerene polymer is modeled as a 3D lattice in which the *b* and *c* lattice constants are chosen to be 28 Å so that there is negligible interaction between any two 1D fullerene polymers in the 3D lattice. Similarly, for monolayer fullerene networks, the *c* lattice constant is set to 24 Å.

The band structures are calculated using the unscreened hybrid functional, in which the Hartree–Fock and PBEsol exchange energies are mixed in a 1:3 ratio along with the full PBEsol correlation energy,^48−50^ because it provides better descriptions of the measured electronic and optical band gaps^14−17^ (for details, see the band gaps obtained at different levels of theory in the Supporting Information). Following this, the transport properties are calculated by utilizing the hybrid-functional eigenenergies and eigenstates in **k**-meshes of 10 × 1 × 1 and 8 × 8 × 1 for 1D and 2D C_60_, respectively, with an interpolation factor of 100. The scattering rates for acoustic deformation potential and ionized impurity scattering are obtained using amset,^51^ with the elastic tensor coefficients computed using the finite differences method^52,53^ and the static dielectric constant computed using density functional perturbation theory.^54^ Excitonic effects are considered using the time-dependent Hartree–Fock calculations, which solve the Casida equation using the eigenenergies and wave function computed from the unscreened hybrid functional as inputs.^55,56^**k**-meshes of 10 × 1 × 1 and 8 × 8 × 1 are used for 1D and 2D C_60_, respectively, with the highest eight valence bands and the lowest eight conduction bands included as the basis to converge the computed exciton eigenenergies within 3 meV.

For the thermodynamics of the hydrogen evolution reaction, geometry optimization consistently leads to the adsorption of a hydrogen atom on the top site for all symmetry irreducible carbon atoms (except those involved in the [2+2] cycloaddition bond that are chemically saturated). To avoid the interactions between hydrogen atoms in neighboring cells, 2 × 1 × 1 and 2 × 2 × 1 supercells are used for 1D and 2D C_60_, respectively, with electronic **k**-point grids of 3 × 1 × 1 and 3 × 3 × 1. All atoms undergo full relaxation, including both the lattice constants and internal atomic coordination. A significant contribution to the free energy of the system is the vibrational energy, and we compute the vibrational energies of both the hydrogen atom and the neighboring carbon atoms within a radius of 2.5 Å of the adsorbed hydrogen atom. The vibration contribution to the free energy at room temperature is computed by vaspkit.^57^ The Gibbs free energy of the initial system is calculated using the energy per C_60_ supercell, the energy of half a hydrogen molecule, and half the vibrational energy associated with a free H_2_. Then, the total energy of intermediate *H is calculated, with the change in vibrational energy between the adsorbate form and the pure form included.

## Supporting Information Available

Dielectric function of 0D, 1D, and 2D qTP2 and 3D *Immm* C_60_, as well as bulk and monolayer MoS_2_ (Figure S1), and calculated band gaps (electronvolts) of bilayer and monolayer qHP, monolayer qTP2, and 1D C_60_ using different levels of theory (Table S1) (PDF)
Transparent Peer Review report available (PDF)
