# Supplementary material for: Boosting Photocatalytic Water Splitting of Polymeric C60 by Reduced Dimensionality from Two-Dimensional Monolayer to One-Dimensional Chain
Source: J Phys Chem Lett. 2023 Dec 21;14(51):11768–73. doi: 10.1021/acs.jpclett.3c02578 (PMC10758114; doi:10.1021/acs.jpclett.3c02578)
Supplement: Supplementary file 1 — jz3c02578_si_001.pdf [file jz3c02578_si_001.pdf]

Supporting Information for "Boosting Photocatalytic Water Splitting of Polymeric C_{60} by Reduced Dimensionality from 2D Monolayer to 1D Chain"

Cory Jones[1] and Bo Peng[2]

[1]*Selwyn College, University of Cambridge, Grange Road, Cambridge CB3 9DQ, United Kingdom*

[2]*Theory of Condensed Matter Group, Cavendish Laboratory, University of Cambridge, J. J. Thomson Avenue, Cambridge CB3 0HE, United Kingdom*[*]

(Dated: November 20, 2023)

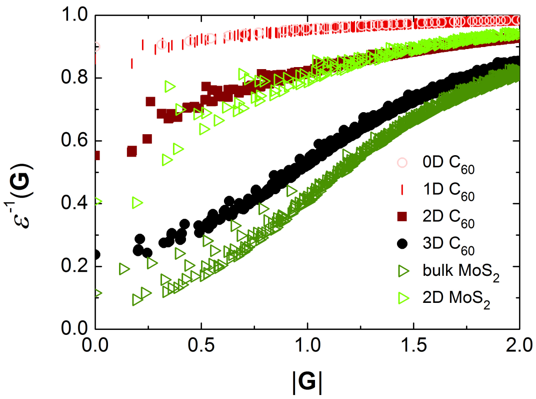

FIG. S1. Dielectric function of 0D, 1D, 2D qTP2 and 3D *Immm* C_{60}, as well as bulk and monolayer MoS_2.

TABLE S1. Calculated band gaps (eV) of bilayer and monolayer qHP, monolayer qTP2 and 1D C_{60} using the semilocal PBEsol functional [1], screened hybrid functional HSEsol [2] and unscreened hybrid functional PBEsol0 [3–5].

	PBEsol	HSEsol	PBEsol0	measured
few-layer qHP C_{60}	0.84	-	2.02	≥ 2.02 [6], ≥ 2.05 [7]
monolayer qHP C_{60}	0.86	1.44	2.12	≥ 1.6 [8]
monolayer qTP2 C_{60}	0.94	1.48	2.18	-
1D C_{60}	1.23	1.81	2.46	-

* bp432@cam.ac.uk

[1] J. P. Perdew, A. Ruzsinszky, G. I. Csonka, O. A. Vydrov, G. E. Scuseria, L. A. Constantin, X. Zhou, and K. Burke, Phys. Rev. Lett. **100**, 136406 (2008).

[2] L. Schimka, J. Harl, and G. Kresse, J. Chem. Phys. **134**, 024116 (2011).

[3] J. P. Perdew, M. Ernzerhof, and K. Burke, J. Chem. Phys. **105**, 9982 (1996).

[4] C. Adamo and V. Barone, J. Chem. Phys. **110**, 6158 (1999).

[5] M. Ernzerhof and G. E. Scuseria, J. Chem. Phys. **110**, 5029 (1999).

[6] E. Meirzadeh, A. M. Evans, M. Rezaee, M. Milich, C. J. Dionne, T. P. Darlington, S. T. Bao, A. K. Bartholomew, T. Handa, D. J. Rizzo, R. A. Wiscons, M. Reza, A. Zangiabadi, N. Fardian-Melamed, A. C. Crowther, P. J. Schuck, D. N. Basov, X. Zhu, A. Giri, P. E. Hopkins, P. Kim, M. L. Steigerwald, J. Yang, C. Nuckolls, and X. Roy, Nature **613**, 71 (2023).

[7] T. Wang, L. Zhang, J. Wu, M. Chen, S. Yang, Y. Lu, and P. Du, Angew. Chem. Int. Ed. **62**, e202311352 (2023).

[8] L. Hou, X. Cui, B. Guan, S. Wang, R. Li, Y. Liu, D. Zhu, and J. Zheng, Nature **606**, 507 (2022).
