# Supplementary material for: Boosting Photocatalytic Water Splitting of Polymeric C60 by Reduced Dimensionality from Two-Dimensional Monolayer to One-Dimensional Chain
Source: J Phys Chem Lett. 2023 Dec 21;14(51):11768–73. doi: 10.1021/acs.jpclett.3c02578 (PMC10758114; doi:10.1021/acs.jpclett.3c02578)
Supplement: Supplementary file 2 — jz3c02578_si_002.pdf [file jz3c02578_si_002.pdf]

jz-2023-02578s.R1

Name: Peer Review Information for "Boosting Photocatalytic Water Splitting of Polymeric C₆₀ by Reduced Dimensionality from 2D Monolayer to 1D Chain"

First Round of Reviewer Comments

Reviewer: 1

Comments to the Author

In this manuscript, the authors studied the photocatalytic performance of 2D qTP and 1D C60 using the state-of-the-art first principles calculations. By reducing the dimension from 2D to 1D, more promising physical/chemical properties are predicted for hydrogen evolution reaction.

I have the following comments/questions for the authors to address:

1. The band gap increases only slightly (2.13 to 2.46 eV) from 2D to 1D. Compared with the huge band gap increasing from bulk MoS2 to monolayer MoS2, the difference in the band gap is rather small. The authors should clarify why the band gap only increases slightly in C60.

2. The authors should compare the calculated band gaps with the measured ones, for example, in [Nature 2022, 606, 507], [Nature 2023, 613, 71] and [Angew. Chem. Int. Ed. 2023, 62, e202311352].

3. The authors only computed the relevant properties for monolayer fullerene networks, while in abstract they cited the Natuer paper on few-layer fullerene. This could create confusion on the systems they studied. The authors should be specific on monolayer fullerene networks in the abstract.

Author's Response to Peer Review Comments:

Reviewer: 1

Recommendation: This paper is publishable subject to minor revisions noted. Further review is not needed.

Answer: We thank the reviewers for their high evaluation of our work and for their detailed comments that help improve our manuscript.

Comments:

In this manuscript, the authors studied the photocatalytic performance of 2D qTP and 1D C_{60} using the state-of-the-art first principles calculations. By reducing the dimension from 2D to 1D, more promising physical/chemical properties are predicted for hydrogen evolution reaction.

We have the following comments/questions for the authors to address:

1. The band gap increases only slightly (2.13 to 2.46 eV) from 2D to 1D. Compared with the huge band gap increasing from bulk MoS_2 to monolayer MoS_2, the difference in the band gap is rather small. The authors should clarify why the band gap only increases slightly in C_{60}.

Answer: We thank the reviewer for noticing the band gap difference between low-dimensional MoS_2 and C_{60}. Because 2D C_{60} has a much lower static dielectric function ($\varepsilon_\infty = 1.8$) than bulk MoS_2 ($\varepsilon_\infty = 8.6$), further reducing its dimension to 1D C_{60} only leads to a tiny change of ε_∞. The ε_∞ of 2D C_{60} chain is only 1.5 times larger than that of 1D C_{60}, whereas the ε_∞ of bulk MoS_2 is more than 3.5 times the ε_∞ of monolayer MoS_2, as shown in Fig. 1. As a result, MoS_2 is much more sensitive to reduced dimension, as their dielectric screening is significantly weakened from bulk to monolayer.

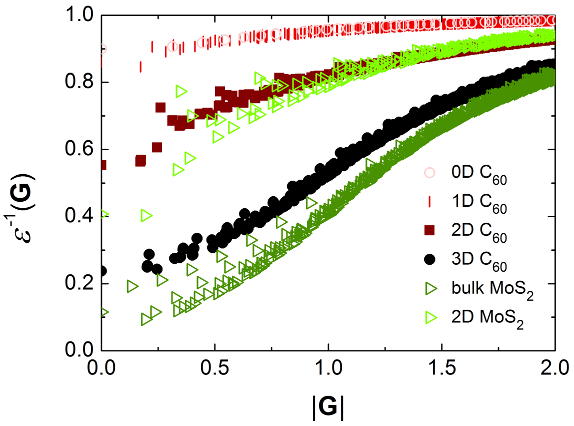

Figure 1. Dielectric function of 0D, 1D, 2D qTP2 and 3D *Immm* C_{60}, as well as bulk and monolayer MoS_2.

We have added Fig. 1 in the Supporting Information and clarified the difference in ε_∞ in the main text, as follows:

Compared to the band gap difference of about 0.6 eV between monolayer and bulk MoS_2,[1] the relatively small band gap difference between 1D and 2D C_{60} of 0.33 eV is due to their similar dielectric screening. The static dielectric function ε_∞ of bulk MoS_2 is more than 3.5 times larger than its monolayer counterpart, whereas the ε_∞ of monolayer qTP2 C_{60} is approximately 1.5 times the ε_∞ of 1D C_{60} chain (for details, see the dielectric function in the Supporting Information).

2. The authors should compare the calculated band gaps with the measured ones, for example, in [*Nature* **2022**, 606, 507], [*Nature* **2023**, 613, 71] and [*Angew. Chem. Int. Ed.* **2023**, 62, e202311352].

Answer: We have compared the computed band gaps with the measured ones in Table 1 and added it to the Supporting Information.

Table 1. Calculated band gaps (eV) of bilayer and monolayer qHP, monolayer qTP2 and 1D C_{60} using the semilocal PBEsol functional ,[2] screened hybrid functional HSEsol [3] and unscreened hybrid functional PBEsol0 .[4–6]

	PBEsol	HSEsol	PBEsol0	measured
few-layer qHP C_{60}	0.84	-	2.02	$\geq$ 2.02 ,[7] $\geq$ 2.05 [8]
monolayer qHP C_{60}	0.86	1.44	2.12	1.6 [9]
monolayer qTP2 C_{60}	0.94	1.48	2.18	-
1D C_{60}	1.23	1.81	2.46	-

3. The authors only computed the relevant properties for monolayer fullerene networks, while in abstract they cited the *Nature* paper on few-layer fullerene. This could create confusion on the systems they studied. The authors should be specific on monolayer fullerene networks in the abstract.

Answer: We thank the reviewer for pointing out the difference between monolayer and few-layer C_{60} in the two synthesis papers. For clarity, we have moved the reference on few-layer fullerene networks in the abstract to the introduction and kept the reference on the monolayer fullerene networks, as follows,

Abstract: Recent synthesis of monolayer fullerene networks [Nature ***2022****, 606, 507] provides new opportunities for photovoltaics and photocatalysis because of their versatile crystal structures for further tailoring of electronic, optical and chemical function.*

...

Introduction: ... Recently synthesised 2D fullerene networks [7,9] *hold great promise for such applications owing to the benefits of (1) a suitable band gap to generate a large amount of electron-hole pairs, (2) high carrier mobility to separate electrons and holes, and (3) appropriate band edges to thermodynamically drive the hydrogen evolution reaction with the help of photoexcited electrons, and the computational predictions based on such microscopic mechanisms* [10] *have been recently confirmed experimentally .*[8]

Additional Questions:
Urgency: Top 10%
Significance: Top 10%
Novelty: Top 10%
Scholarly Presentation: Top 10%
Is the paper likely to interest a substantial number of physical chemists, not just specialists working in the authors' area of research?: Yes

References

1. Padilha, J. E., Peelaers, H., Janotti, A. & Van de Walle, C. G. Nature and evolution of the band-edge states in mos_2: From monolayer to bulk. *Phys. Rev. B* **90**, 205420 (2014).
2. Perdew, J. P. *et al.* Restoring the Density-Gradient Expansion for Exchange in Solids and Surfaces. *Phys. Rev. Lett.* **100**, 136406 (2008).
3. Schimka, L., Harl, J. & Kresse, G. Improved hybrid functional for solids: The hsesol functional. *J. Chem. Phys.* **134**, 024116 (2011).
4. Perdew, J. P., Ernzerhof, M. & Burke, K. Rationale for mixing exact exchange with density functional approximations. *J. Chem. Phys.* **105**, 9982–9985 (1996).
5. Adamo, C. & Barone, V. Toward reliable density functional methods without adjustable parameters: The pbe0 model. *J. Chem. Phys.* **110**, 6158–6170 (1999).
6. Ernzerhof, M. & Scuseria, G. E. Assessment of the perdew-burke-ernzerhof exchange-correlation functional. *J. Chem. Phys.* **110**, 5029–5036 (1999).
7. Meirzadeh, E. *et al.* A few-layer covalent network of fullerenes. *Nature* **613**, 71–76 (2023).
8. Wang, T. *et al.* Few-layer fullerene network for photocatalytic pure water splitting into h_2 and h_2o_2. *Angew. Chem. Int. Ed.* **62**, e202311352 (2023).
9. Hou, L. *et al.* Synthesis of a monolayer fullerene network. *Nature* **606**, 507–510 (2022).
10. Peng, B. Monolayer fullerene networks as photocatalysts for overall water splitting. *J. Am. Chem. Soc.* **144**, 19921–19931 (2022).
